# Annual Versus Patient‐Initiated Follow‐Up in Sleep Apnoea Care: A Quasi‐Experimental Study

**DOI:** 10.1111/jsr.70350

**Published:** 2026-05-20

**Authors:** Karin Jeppesen, Donna Lykke Wolff, Sofie Ronja Petersen, Asbjørn Kørvel‐Hanquist, Jens Pilgaard Jacobsen, Eva Kirkegaard Kiær, Poul Jørgen Jennum, Preben Homøe, Helene Skjøt‐Arkil

**Affiliations:** ^1^ Department of Ear, Nose and Throat Surgery University Hospital of Southern Denmark Sønderborg Denmark; ^2^ Department of Regional Health Research University of Southern Denmark Odense Denmark; ^3^ Department of Clinical Research University Hospital of Southern Denmark Aabenraa Denmark; ^4^ Department of Otorhinolaryngology and Maxillofacial Surgery Zealand University Hospital Køge Denmark; ^5^ Department of Clinical Medicine, Faculty of Health Sciences University of Copenhagen Copenhagen Denmark; ^6^ Department of Otorhinolaryngology, Head and Neck Surgery, and Audiology Copenhagen University Hospital Copenhagen Denmark; ^7^ Danish Center for Sleep Medicine Department of Clinical Neurophysiology Glostrup Denmark; ^8^ Department of Health Technology Technical University of Denmark Kongens Lyngby Denmark

**Keywords:** adherence, cost analysis, remote monitoring, sleep‐disordered breathing, telemonitoring, virtual care

## Abstract

Obstructive sleep apnoea affects nearly 1 billion people worldwide, placing increasing pressure on healthcare systems. Continuous positive airway pressure (CPAP) therapy is often the standard treatment, and efficient follow‐up strategies are needed to maintain adherence and optimise hospital resources. This study aimed to evaluate whether CPAP follow‐up, conducted through traditional scheduled annual in‐person or patient‐initiated contacts, was most effective based on adherence rates, contact type and frequency, and associated costs. A quasi‐experimental design was used to compare CPAP follow‐up practices in two sleep clinics. The primary outcome was adherence after 2 years, assessed using telemonitoring data from the CPAP devices. Registry data were used to assess contact frequency and type, as well as diagnosis‐related group tariffs to estimate hospital contact costs. Adherence rates were 80% in both sleep clinics after 2 years. Patients with scheduled annual contacts had an average of 4.09 (SD 1.63) interactions, primarily in‐person, whereas those with patient‐initiated contacts had 5.09 (SD 3.01), most of which were virtual. The cost‐minimisation analysis revealed no statistically significant difference in overall costs between the two follow‐up models, with a mean of 1210 EUR (IQR 1129–1614) in the scheduled contact group and 1076 EUR (IQR 700–1507) in the patient‐initiated group. To our knowledge, this is the first study to examine the impact of replacing scheduled CPAP follow‐up contacts with patient‐initiated contacts. Given the comparable adherence rates and costs, we conclude that scheduled annual in‐person follow‐up may be considered for replacement with a more flexible, virtual, patient‐driven follow‐up model.

## 1 Introduction

### 1.1 Background

Worldwide, approximately 1 billion patients are affected by obstructive sleep apnoea (OSA), and the prevalence is rising, partly due to an ageing population and the global obesity epidemic (Benjafield et al. 2019; Garvey et al. 2015). Continuous positive airway pressure (CPAP) therapy is the standard first‐line treatment for OSA patients (Patil et al. 2019), and adherence to and duration of CPAP use are positively correlated with improved outcomes (Sánchez‐de‐la‐Torre et al. 2023). Untreated OSA is associated with an increased risk of cardiovascular, cerebrovascular, and metabolic diseases, mortality and traffic accidents, as well as a decreased quality of life (Højager et al. 2023; Jennum et al. 2013; Pépin et al. 2022; Torres et al. 2024).

The increasing number of patients with OSA poses a significant burden on healthcare resources (Lugo et al. 2019) due to the rising number of patients who need treatment, and correlation with severe comorbidities and days away from the labour market (Jennum et al. 2025; Marquina et al. 2022). Therefore, healthcare systems are struggling to keep pace with the rising prevalence of OSA (Isetta et al. 2015; Turino et al. 2017), resulting in longer waiting lists and limited consultation time per patient. Consequently, new approaches for managing the follow‐up of CPAP‐treated patients are required to ensure that patients receive the highest standard of care, while simultaneously promoting efficiency and optimal resource utilisation within the healthcare system (Pépin et al. 2025, 2024).

Studies have explored CPAP adherence and follow‐up methods with mixed results. Generally, they report that virtual contacts, delivered via video or telephone, are as effective as in‐person contacts (Chen et al. 2020; Contal et al. 2021; Jones et al. 2024; Simonelli et al. 2024). Telemonitoring has been demonstrated to be as effective as in‐person follow‐up in relation to CPAP adherence, symptom improvement, and cost‐effectiveness (Alsaif et al. 2022; Isetta et al. 2015; Niu et al. 2023; Turino et al. 2017; van der Kleij et al. 2024). These studies compared different follow‐up models, where the telemonitoring groups received an equal or higher frequency of contacts than the standard follow‐up. To our knowledge, no studies have investigated adherence to CPAP therapy, the number of additional contacts, and costs in a follow‐up model based solely on patient‐initiated contacts.

High adherence to CPAP within the first months after therapy initiation has been associated with long‐term high adherence (Jeppesen, Kørvel‐Hanquist, et al. 2025b; Yi et al. 2022). Poor follow‐up procedures can negatively influence adherence. Therefore, it is necessary to determine which types of follow‐up practices can be modified or removed, in order to treat more OSA patients with the same effect and resources.

This study will evaluate current follow‐up practices for patients with high adherence to CPAP in two sleep clinics to identify the most effective approach to maintain optimal patient outcomes and resource utilisation.

### 1.2 Aim

This study aimed to investigate whether there were differences in adherence, the number and type of patient contacts, and associated costs between patients receiving CPAP follow‐up with scheduled annual in‐person compared to patient‐initiated contacts.

## 2 Methods

### 2.1 Study Design and Setting

We used a quasi‐experimental design (Schweizer et al. 2016) to conduct a comparative analysis of two clinical approaches: Hospital A, with scheduled annual contacts, versus Hospital B, with patient‐initiated contacts only after established CPAP therapy. The study was reported in accordance with the TREND (Transparent Reporting of Evaluations with Nonrandomized Designs) statement (Des Jarlais et al. 2004).

Participants were allocated to either hospital based on residential catchment area, rather than random assignment, resulting in a non‐equivalent group comparison. OSA patients were included from the Sleep Clinic at University Hospital of Southern Denmark (Hospital A) in Sønderborg, Region of Southern Denmark and the Sleep Clinic at Zealand University Hospital (Hospital B) in Køge, Region Zealand. Hospital A and B provide services to a catchment area of approximately 225,000 and 855,000 individuals, respectively, representing a combined coverage of 18% of the Danish population. We collected data from medical records, tele‐monitored CPAP therapy and national registers. The study period was defined as the day the Sleep Clinics began tele‐monitored CPAP therapy (1 January 2020 and 1 January 2015, for Hospitals A and B, respectively) until 31 December 2022. Patients were followed for 2 years within this study period.

### 2.2 Participants

Patients diagnosed with OSA were referred to the sleep clinics. The diagnosis could be made by either a general otolaryngologist or in a sleep clinic. In both cases, a medical examination by a doctor and cardiorespiratory monitoring, or partial polygraphy, had been performed to confirm the diagnosis. In both sleep clinics, patients were offered CPAP as first‐line treatment. Patients were primarily initiated on auto‐adjusting positive airway pressure (APAP) devices, with pressure settings typically ranging from 4 to 20 cmH_2_O. Expiratory pressure relief (EPR) was enabled when clinically indicated. Included in this study were patients who achieved high adherence to CPAP therapy within the first 3 months and were transitioned to a standard follow‐up programme, as illustrated in Figure 1. Patients with lower adherence received individualised follow‐up depending on the specific issues they experienced with CPAP therapy and were not included in this study.

**FIGURE 1:**
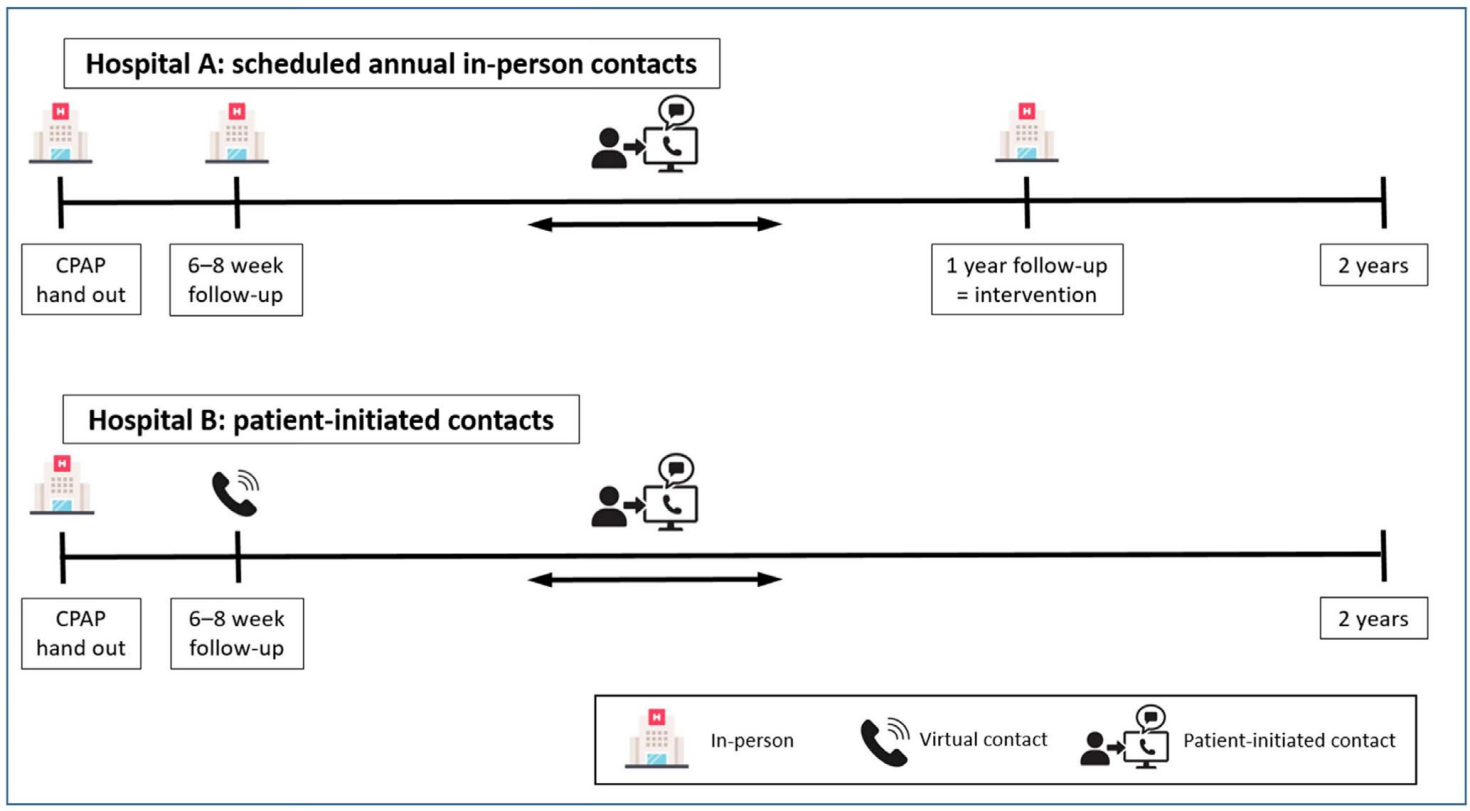
Standard follow‐up programme for patients who achieved high adherence at 3 months in the respective hospitals. CPAP, continuous positive airway pressure.

The study population consisted of newly diagnosed adult OSA patients (≥ 18 years of age) who initiated CPAP therapy with a device connected to telemonitoring technology in one of the two sleep clinics. CPAP treatment is free of charge in Denmark and is collectively paid via tax. There was no proactive monitoring, although telemonitoring could perform remote technical adjustments if patients contacted the sleep clinics. To be included, patients were required to be incident OSA patients and have achieved high adherence within the first 3 months after CPAP initiation and to have at least 2 years of follow‐up data available. Patients were excluded if the CPAP device was returned within 2 years.

### 2.3 Variables and Data Sources

The primary outcome variable was CPAP adherence after 2 years. The CPAP devices recorded daily usage data and stored data with a cloud‐based solution by ResMed AirView. Patients were grouped into two levels of adherence: high and low/no adherence. The definition of high adherence was based on the Center for Medicare and Medicaid Services (CMS) criterion, which requires CPAP use of ≥ 4 h per night for ≥ 70% of the observation period (Kribbs et al. 1993). Missing adherence data were interpreted as nights where CPAP was not used and were categorised as zero‐hour use.

Hospital contacts were defined as all interactions between the patient and the sleep clinic staff. In the Danish healthcare system, patients do not contact CPAP providers or device suppliers directly; all equipment‐related requests and support are managed through the sleep clinics.

The secondary outcome, Hospital contacts, was divided into the following four categories:

- In‐person contact: any contact where the patient met in person at the sleep clinic. If needed, CPAP equipment could be provided at the visit.
- Virtual contact with AirView: contact made by telephone, email, or online video meeting, where the patient's AirView profile was reviewed and used for the consultation. If needed, CPAP equipment could be provided.
- Virtual contact without AirView: contact to achieve supervision of CPAP therapy, but where AirView was not used and no coding for CPAP equipment was performed.
- Requests for CPAP equipment: Virtual contact to achieve CPAP equipment only.

The annual follow‐up visit was defined as an in‐person contact at approximately 14 months after initiation of CPAP, corresponding to 12 months after the 6–8‐week follow‐up contact. Contacts occurring before 7 months or after 21 months fell outside the definition of annual follow‐up visits.

Hospital costs were based on Diagnosis‐Related Group (DRG) tariffs. We applied the 2025 DRG tariffs, adjusted to 2022 price levels, as the original 2022 tariffs did not accurately reflect actual resource consumption.

Co‐variables, including age, sex, Apnoea–Hypopnea Index (AHI) (Kapur et al. 2017), Oxygen Desaturation Index (ODI), and Epworth Sleepiness Scale (ESS) (Rotenberg et al. 2016), scores were obtained from the Danish National Patient Registry (Lynge et al. 2011). Socioeconomic data were extracted from the Civil Registration System (Schmidt et al. 2014), the Income Registry (Baadsgaard and Quitzau 2011), and the Education Registry (Jensen and Rasmussen 2011). Co‐variables were recorded from the date the CPAP device was issued. All data were combined using a unique personal identification number (CPR) provided for all Danish citizens (Schmidt et al. 2014).

### 2.4 Intervention and Assignment Method

The standard follow‐up programme varied between the two sleep clinics in terms of type and frequency of scheduled contacts, as illustrated in Figure 1. Hospital A offered a scheduled annual in‐person contact, which is considered the intervention in this study. In contrast, Hospital B offered follow‐up only at the patients' request after the initial 6–8‐week contact. The patients were not randomly assigned to the two hospitals; instead, they were allocated to each sleep clinic (Hospital A or Hospital B) based on their residential address within the respective catchment areas.

### 2.5 Study Size

The sample size consisted of all available observations from patients who fulfilled the inclusion criteria. As the primary analysis was conducted on aggregated weekly adherence rates across all patients, the unit of measurement was ‘adherence rate per week’. This approach reduces the impact of variation in patient numbers between clinics on the overall analysis.

### 2.6 Statistical Methods

Descriptive methods were used to present the baseline characteristics of the study population. Normally distributed numerical variables were presented as means with standard deviations (SD), while skewed variables, including economic data, were reported as medians with interquartile ranges (IQR). Categorical variables were reported as absolute and relative frequencies.

To evaluate the effectiveness of the planned annual in‐person contact, we applied interrupted time series analysis (ITSA) based on two periods (pre‐ and post‐follow‐up visits) and two groups (hospitals). The pre‐ and post‐intervention periods for Hospital A were defined as the maximum number of weeks with sufficient data, as confirmed by sensitivity analysis. Due to no planned follow‐up contact in the Hospital B group, we set the split date (pre/post) to 15 months after CPAP initiation. The adherence proportion (percentage of patients in the high adherence group) was aggregated at the population level on a weekly basis. To ensure the validity of this comparison, a two‐arm interrupted time series analysis (ITSA) (Linden 2015) was performed with data following an AR(2)‐process. We also investigated the pre‐ and post‐visit difference in a one‐arm ITSA, using data from Hospital A only.

For each contact type, we compared the number of contacts between hospitals using Poisson and logistic regression models. These models were adjusted for the following confounders: sex, age, AHI and ESS. Model assumptions were assessed using residual plots. Additionally, we presented the proportion of hospital contacts in both groups and conducted a cost‐effectiveness analysis or, if outcomes did not differ, a cost‐minimisation analysis.

Handling of missing data and erroneous data: For some patients, the use of CPAP for more than 24 h per day was reported in the ResMed AirView database. We replaced implausible values with the average of the available correct observations for these data points. For the variables AHI, ODI, and ESS, missing values could be present due to administrative reasons or because registrations fell outside the possible range. Due to the nature of the data and study design, values were assumed to be missing at random.

All estimates were reported with a 95% confidence interval (CI), and a 5% significance level was used for two‐sided tests. Statistical analyses were performed using Stata (College Station, Texas, USA: StataCorp LLC).

## 3 Ethical Considerations

The disclosure of patient record information without informed consent is based on a regional council approval according to Section 46, part 2 of the Health Act (journal number 22/60970). Approval for access to the electronic patient records was obtained from the management of the two involved hospitals, and the processing of personal data was notified to and approved by the Region of Southern Denmark, and listed in the internal record (journal number 23/4511) cf. art 30 of The EU General Data Protection Regulation. Approval by the Ethics Committee or informed consent is not required for register‐based studies according to Danish law.

## 4 Results

This study included 742 newly diagnosed OSA patients who demonstrated high adherence to CPAP therapy 3 months after initiation (Figure 2). Of these, 174 (23%) were from Hospital A, where follow‐up procedures include annual in‐person contact, and 568 (77%) were from Hospital B, where follow‐up is at the patient's request only.

**FIGURE 2:**
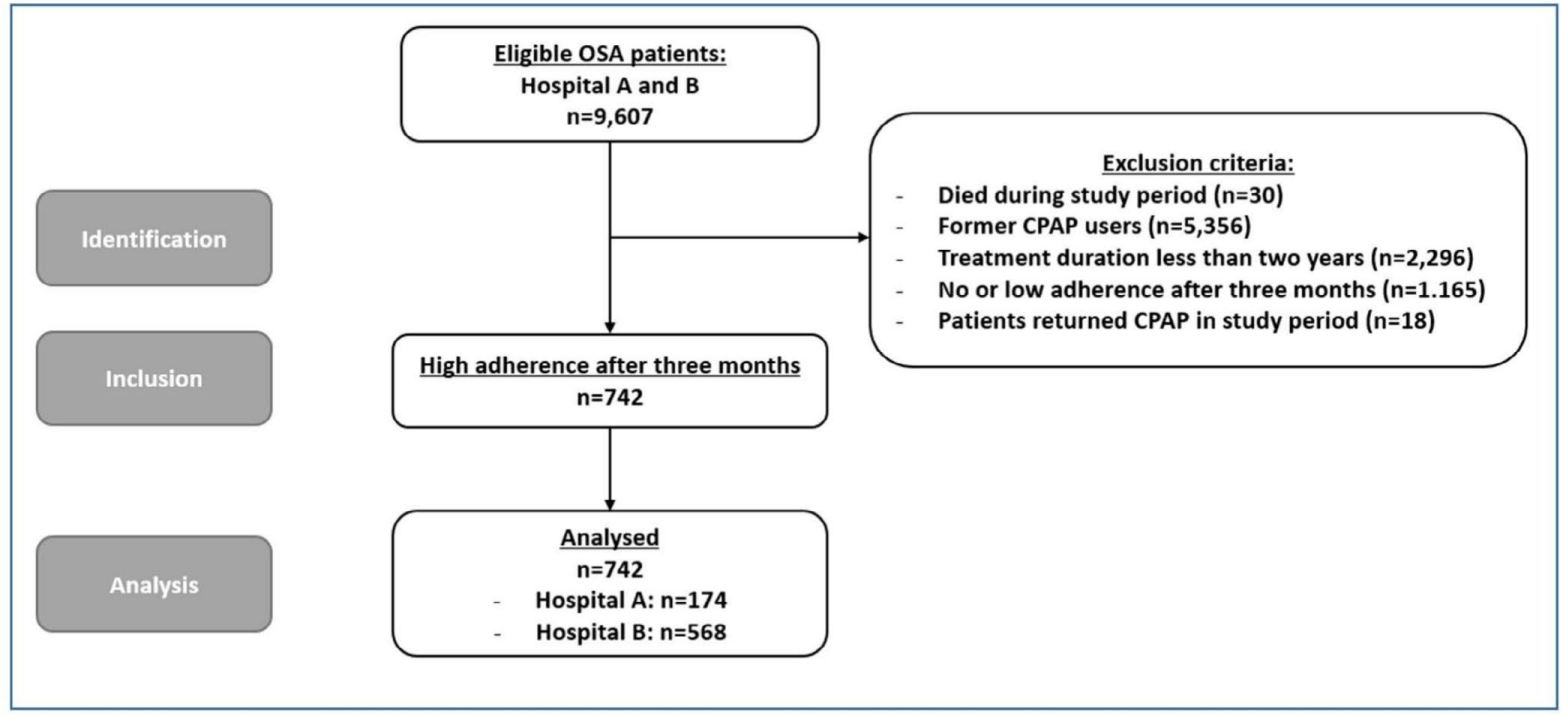
Study enrolment flow chart. CPAP, continuous positive airway pressure; OSA, obstructive sleep apnoea.

Baseline characteristics are shown in Table 1. Although no formal statistical comparison was made, inspection of Table 1 suggests that patients in Hospital B had a higher pre‐CPAP AHI compared to those in Hospital A, and Hospital A had a higher proportion of immigrants and descendants from other countries. The majority of immigrants came from Western European countries, which aligns with the presence of German patients living in Denmark, likely due to the hospital's proximity to the German border. Elsewhere, the two groups were very similar.

**TABLE 1:** Patient characteristics at the time of OSA diagnosis.

		Hospital A	Hospital B	Total
		*n* = 174 (23%)	*n* = 568 (77%)	*n* = 742
Age		56.3 (12.4)	56.0 (11.8)	56.1 (11.9)
Sex	Male	114 (65.5%)	412 (72.5%)	526 (70.9%)
	Female	60 (34.5%)	156 (27.5%)	216 (29.1%)
AHI pre‐CPAP	AHI < 15	17 (9.8%)	16 (2.8%)	33 (4.4%)
	15 ≤ AHI < 30	55 (31.6%)	119 (21.0%)	174 (23.5%)
	30 ≤ AHI	77 (44.3%)	340 (59.9%)	417 (56.2%)
	Missing	25 (14.4%)	93 (16.4%)	118 (15.9%)
ODI pre‐CPAP	ODI < 15	13 (7.5%)	31 (5.5%)	44 (5.9%)
	15 ≤ ODI < 30	58 (33.3%)	131 (23.1%)	189 (25.5%)
	30 ≤ ODI	79 (45.4%)	313 (55.1%)	392 (52.8%)
	Missing	24 (13.8%)	93 (16.4%)	117 (15.8%)
ESS pre‐CPAP	Normal daytime sleepiness	40 (23.0%)	105 (18.5%)	145 (19.5%)
	Mild daytime sleepiness	16 (9.2%)	43 (7.6%)	59 (8.0%)
	Moderate daytime sleepiness	61 (35.1%)	190 (33.5%)	251 (33.8%)
	Severe daytime sleepiness	26 (14.9%)	118 (20.8%)	144 (19.4%)
	Missing	31 (17.8%)	112 (19.7%)	143 (19.3%)
Cohabitation status	Living alone	40 (23.0%)	126 (22.2%)	164 (22.2%)
	Cohabitating	134 (77.0%)	442 (77.8%)	576 (77.8%)
Ethnicity	Danish	157 (90.2%)	552 (97.2%)	709 (95.6%)
	Immigrants and descendants	17 (9.8%)	16 (2.8%)	33 (4.4%)
Occupation	Affiliated with the labour market	100 (57.5%)	356 (62.7%)	456 (61.5%)
	Ongoing education	0 (0.0%)	6 (1.1%)	6 (0.8%)
	Unemployed, welfare payment or other	18 (10.3%)	51 (9.0%)	69 (9.3%)
	Early retirement	13 (7.5%)	28 (4.9%)	41 (5.5%)
	Retirement	43 (24.7%)	127 (22.4%)	170 (22.9%)
Disposable income	Missing or low tertile^a^	64 (36.8%)	189 (33.3%)	253 (34.1%)
	Middle tertile	65 (37.4%)	181 (31.9%)	246 (33.2%)
	High tertile	45 (25.9%)	198 (34.9%)	243 (32.7%)
Education	Not known and lower secondary^a^	42 (24.1%)	108 (19.0%)	150 (20.2%)
	Upper secondary	86 (49.4%)	322 (56.7%)	408 (55.0%)
	Post‐secondary	46 (26.4%)	138 (24.3%)	184 (24.8%)

Abbreviations: AHI, apnoea–hypopnea index; BMI, body mass index; CPAP, continuous positive airway pressure; ESS, Epworth Sleepiness Scale; ODI, oxygen desaturation index; OSA, obstructive sleep apnoea.

^a^ Due to data protection regulations, categories with fewer than five individuals (e.g., missing or unknown values) were combined with adjacent groups to ensure anonymity.

Data are presented as the mean (SD) for continuous measures and as *n* (%) for categorical measures.

Figure 3 shows that in‐person contacts at Hospital A occurred approximately 2 months later than expected. The majority of patients had a contact 16 months after initiation of CPAP therapy, rather than the anticipated 14 months. The curves for adherence illustrate that around 80% of the entire study cohort had high adherence after 2 years of CPAP therapy, meaning that 20% of the patients were no longer highly adherent to CPAP in any of the hospitals. In the Hospital A cohort, 89% attended a follow‐up visit, compared to 21% in the Hospital B cohort, within 7–21 months after CPAP initiation.

**FIGURE 3:**
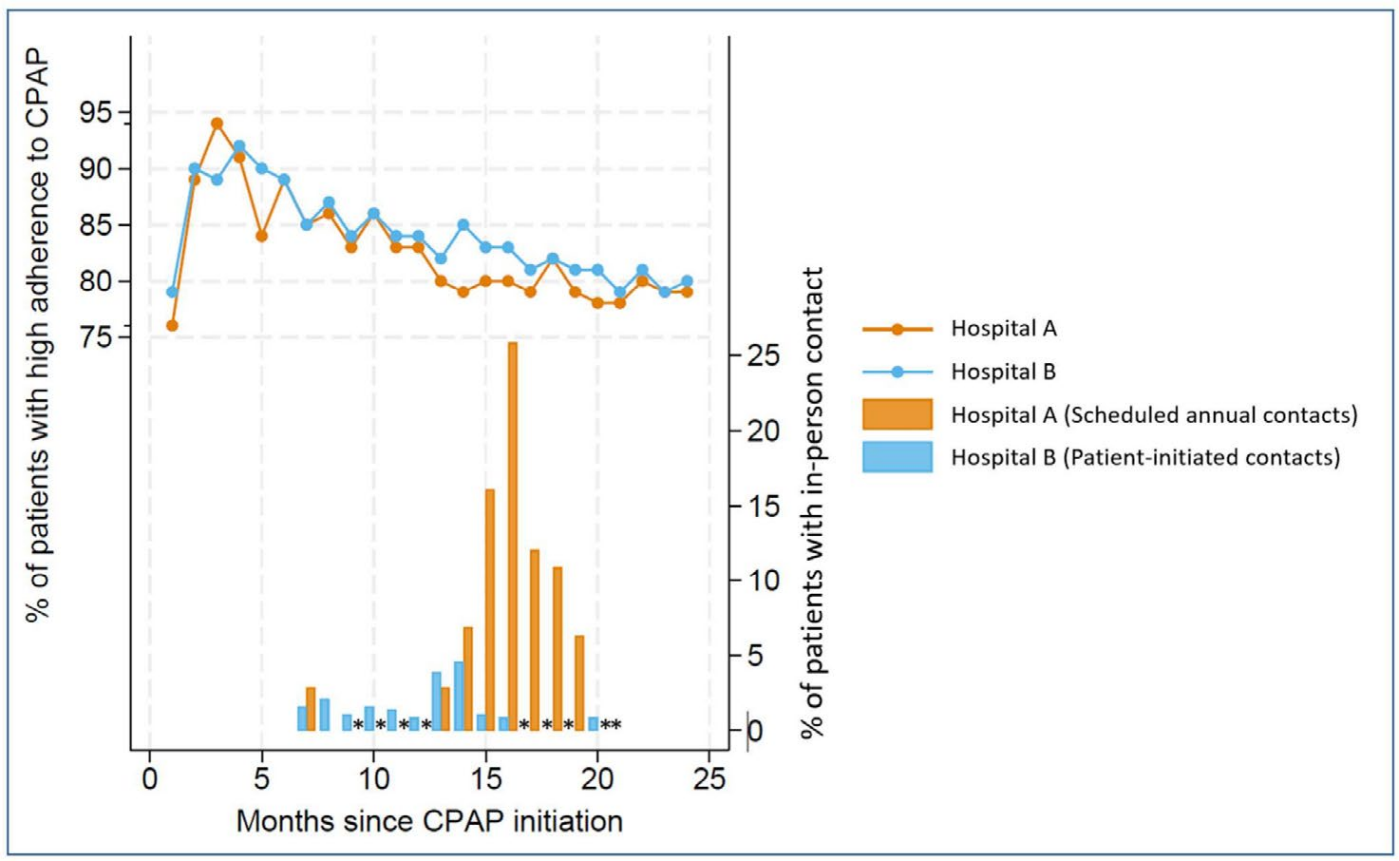
Adherence over time distributed on scheduled annual versus patient‐initiated contacts. * According to the General Data Protection Regulation in Denmark, patient counts below five must not be visualised in figures in register‐based studies. Therefore, groups with ≤ 5 patients are not shown as bars but are marked with an asterisk *in the figure. In total, nine bars were suppressed (counts between 1 and 4) and indicated with an asterisk. CPAP: continuous positive airway pressure.

The ITSA (Figure 4) illustrates the proportion of patients who maintained high adherence in the two hospitals before and after the intervention. This intervention corresponds to in‐person contact for Hospital A and is fictitious for Hospital B, assumed to be at the median date of follow‐up visit for Hospital A. There is no significant difference between the two hospitals (*p* = 0.49).

**FIGURE 4:**
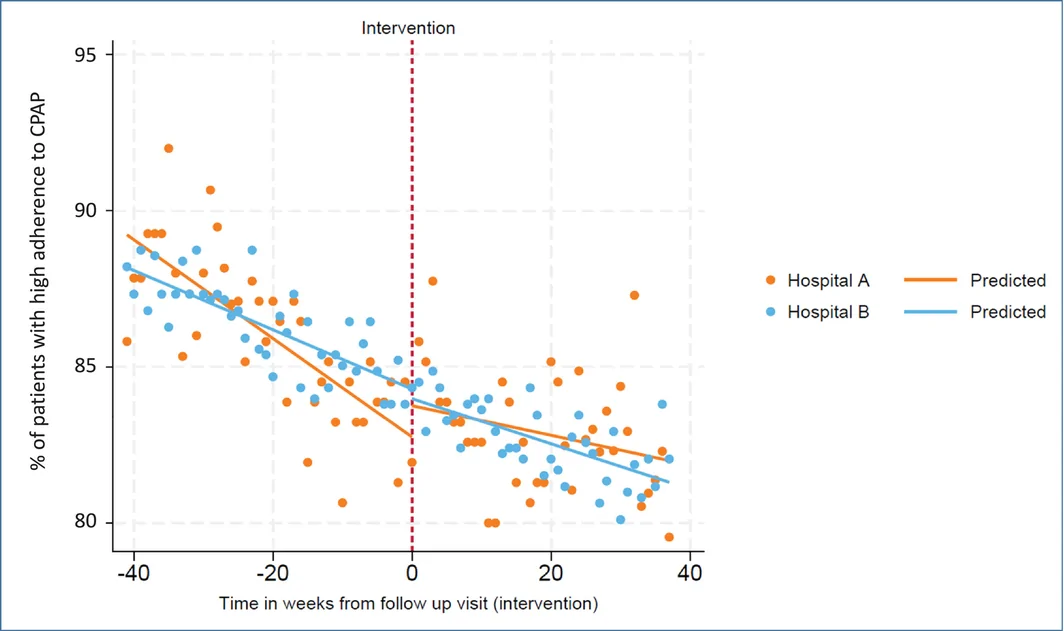
Interrupted time series analysis.

The analysis was performed given that we have adherence data spanning 2 years of follow‐up for each patient, and the annual in‐person contact typically occurs around month 16. However, it varies; therefore, we conducted a sensitivity analysis applied only to Hospital A to determine the optimal number of weeks post‐intervention for data presentation. Our findings (Data S1) indicate that by week 37 post‐intervention, over 57% of the patients still had available data and were thus included in the ITSA‐analysis.

Figure 5 illustrates that most patients at Hospital A had a total of three contacts with the sleep clinic during the study period. In contrast, most patients from Hospital B had six or more contacts. Patients from Hospital A had minimal additional contacts beyond the scheduled in‐person contacts. Conversely, patients from Hospital B typically had one in‐person contact, with subsequent contacts conducted virtually. Apart from a routine follow‐up visit 6–8 weeks after CPAP initiation, the remaining interactions were patient‐initiated. This pattern aligns with the standard follow‐up protocol outlined in Figure 1.

**FIGURE 5:**
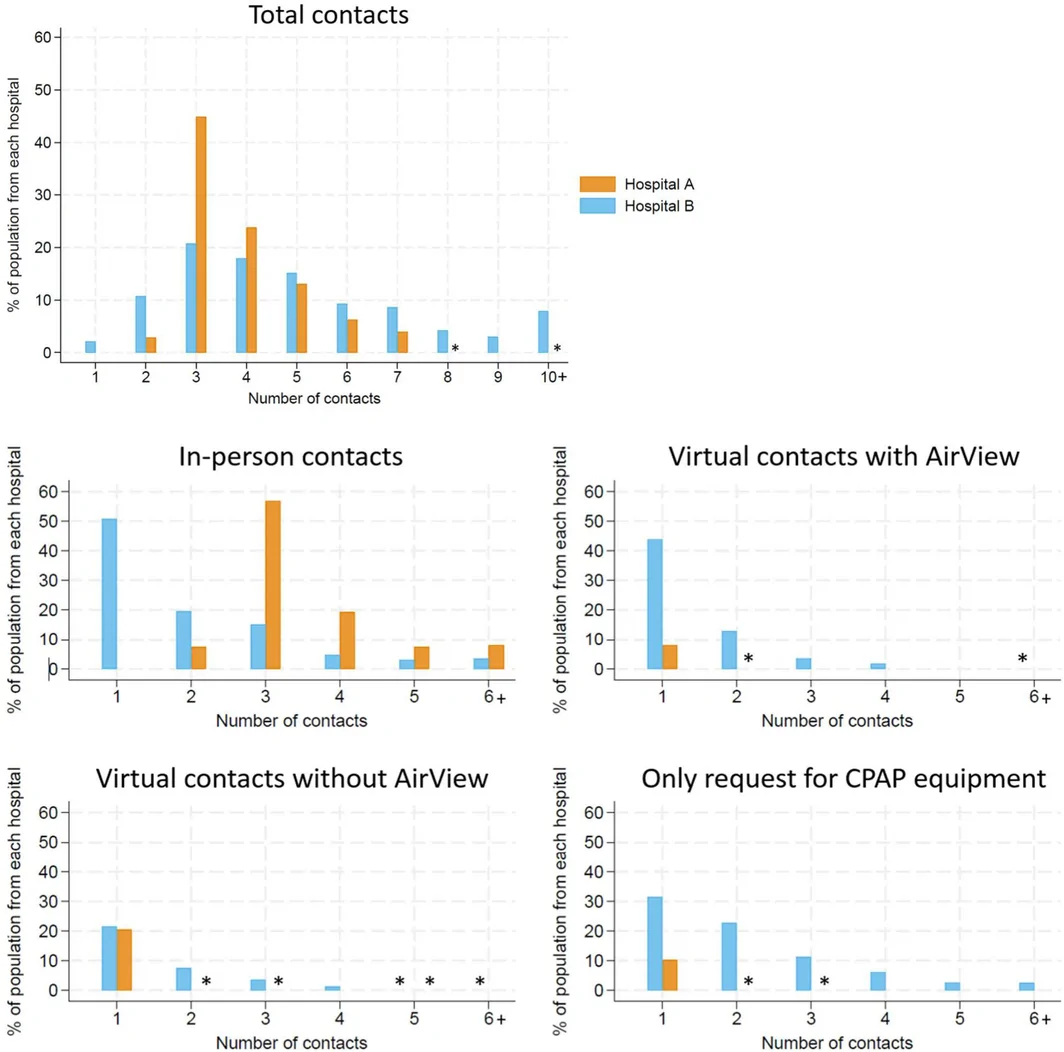
Types of contacts to the sleep clinics. * According to the General Data Protection Regulation in Denmark, patient counts below five must not be visualised in figures in register‐based studies. Therefore, groups with ≤ 5 patients are not shown as bars but are marked with an asterisk *in the figure. CPAP, continuous positive airway pressure.

The total average cost per patient is reduced by 11% in Hospital B (1076 EUR/patient) compared to Hospital A (1210 EUR/patient). These costs were calculated solely based on DRG tariffs, excluding the patients' time and loss of working hours in this analysis. A sensitivity analysis was conducted on total cost minimisation (Data S2). The analysis demonstrated that only a 5% reduction in the DRG tariff for virtual costs would result in a significant difference in the average total costs between the two hospitals.

## 5 Discussion

Scheduled annual in‐person contacts did not influence adherence to CPAP therapy when compared to patient‐initiated contact. Adherence patterns were consistent despite follow‐up type, with 80% of patients in both groups remaining adherent 2 years after initiating CPAP therapy. On average, patients at Hospital A received three planned in‐person contacts and one additional (primarily virtual), whereas patients at Hospital B had two planned contacts (one in‐person and one virtual) and three additional patient‐initiated contacts, most of which were virtual and no significant difference in adherence. The cost‐minimisation analysis revealed that follow‐up models at Hospital B were associated with non‐significantly lower overall costs compared to Hospital A.

To our knowledge, this is the first study to investigate the effects of removing CPAP follow‐up contacts. While several previous studies have examined changes in consultation types, timing, or frequency (Alsaif et al. 2022; Isetta et al. 2015; Lugo et al. 2019; Turino et al. 2017; van der Kleij et al. 2024), none have addressed the consequences of replacing scheduled in‐person follow‐ups with patient‐initiated contact.

### 5.1 Adherence

Our adherence results are consistent with previous studies, which demonstrate no significant difference in CPAP adherence between telemedicine‐based and standard care follow‐up models (Isetta et al. 2015; Lugo et al. 2019; Turino et al. 2017; van der Kleij et al. 2024). In this context, Hospital A may be considered representative of standard care, while Hospital B reflects a telemedicine‐oriented model, given that most patient‐initiated contacts were virtual. A key distinction, however, is that telemonitoring in our study was not used proactively to enhance adherence; it was used solely for remote technical support when patients contacted the clinic.

Notably, we observed that out of included patients with high adherence within the first 3 months, 20% of patients in both hospitals experienced low or no adherence 2 years after CPAP initiation. These patients were not actively identified during routine care and followed the standard clinical pathway, highlighting the potential value of a prospective study with targeted follow‐up in this high‐risk group. This subgroup of low adherent patients may benefit from a more dynamic follow‐up approach, such as proactive telemonitoring or AI‐supported monitoring systems capable of generating alerts in response to reduced adherence, elevated residual AHI, or excessive mask leak, like in the study of Drigo et al. (2025) who showed an adherence rate of 87% five years after CPAP initiation using this approach. It is unclear whether Drigo et al.'s 87% adherence was due to telemonitoring, clinical engagement, or cohort motivation. Their prospective design may have influenced adherence behaviour, whereas this present study was observational and conducted without patient awareness, thereby reflecting a more naturalistic, real‐world setting.

### 5.2 Type and Frequency of Contacts

The average number of patient contacts was significantly higher at Hospital B compared to Hospital A. In Hospital A, most patients had three in‐person contacts and one additional contact during the 2‐year study period (Figure 5). This pattern suggests that CPAP equipment was typically ordered during scheduled visits, as very few patients contacted the clinic solely for supply requests. In contrast, patients at Hospital B had an average of five contacts. The first two were scheduled, while subsequent interactions were initiated by the patient. Initial contacts could be in‐person or virtual, followed by mostly virtual contacts or equipment requests.

Previous studies show practice telemedicine increases contact frequency (Alsaif et al. 2022; Isetta et al. 2015; Lugo et al. 2019; Turino et al. 2017; van der Kleij et al. 2024). For example, Isetta et al. (2015) found that patients in a telemedicine group had more additional visits than those receiving standard care, a finding also observed in our study. However, as highlighted by Pépin et al. (2024), there is still no consensus on the optimal number or timing of in‐person versus virtual follow‐ups to ensure optimal outcomes and adherence. This underscores the importance of identifying which follow‐up contacts can be delegated to other healthcare professionals, shifted to more time‐efficient formats, or potentially eliminated, which was one of the scopes of this study.

Given the increasing number of OSA patients and limited healthcare resources, it is relevant to consider whether certain OSA subgroups could have follow‐up contacts that are less resource‐demanding (Edwards et al. 2019), such as those conducted by other healthcare professionals. Simonelli et al. (2024) found that CPAP adherence was independent of whether follow‐up involved a physician or another professional group, and regardless of whether the contact was virtual or in‐person. It may be worth exploring whether routine tasks, such as CPAP equipment renewal, could be managed by administrative staff or AI chatbots. Similarly, AI‐driven software initially could monitor adherence, with trained staff intervening only when issues arise. Pépin et al. (2024) have suggested that primary care could manage follow‐up for stable OSA patients with high adherence and few comorbidities, as they may have a lower risk of CPAP termination. However, in our study 20% were non‐adherent after 2 years. Therefore, we argue that discharging such patients from specialist care may be premature. A safer approach could involve AI‐based monitoring of adherence, mask leak, and residual AHI, with OSA‐trained staff addressing any emerging issues. For patients who maintain high adherence without complications, a simplified process for obtaining CPAP equipment may be sufficient, potentially reducing the need for further follow‐up.

#### 5.2.1 Responsibility

This raises an important question: Who should be responsible for responding to abnormal results from telemonitored CPAP devices? In the present study, we assigned this responsibility to the patients, encouraging them to contact the clinic when needed. To support this, we ensured easy access via telephone or email, and patients were also offered an app linked to their CPAP device, allowing them to monitor their therapy and reach out to the sleep clinic if necessary. This approach led to an increased number of total contacts compared to standard follow‐up; however, many of these were simple requests for CPAP equipment, which were easily managed (Pépin et al. 2024).

Studies involving routine or proactive contact based on CPAP data (Alsaif et al. 2022; Isetta et al. 2015; Lugo et al. 2019; Turino et al. 2017; van der Kleij et al. 2024) offer a more structured approach, allowing clinics to schedule follow‐ups rather than relying on open availability. However, this model may result in wasted resources if patients do not respond when health care professionals contact them. In contrast, when patients are responsible for initiating contact, they take ownership of their therapy, which is an important factor in maintaining adherence in chronic disease management (Whear et al. 2013). Nevertheless, placing full responsibility on the patient may not be sufficient. A more balanced, cooperative model between the patient and the clinic may be needed to ensure continuous high adherence.

### 5.3 Cost‐Minimisation Analysis

As adherence to CPAP, the primary outcome, was found to be both statistically and clinically equivalent between the two hospitals, we proceeded with a cost‐minimisation analysis (Table 2), which was conducted in accordance with standard health economic methodology.

**TABLE 2:** Average number of contact types per patient and cost‐minimisation analysis.

*N*	Hospital A	Hospital B	Test
	174 (23%)	568 (77%)	
Total contacts (count)^d^	4.09 (1.63)	5.09 (3.01)	**< 0.001** ^a^
In‐person contact			
Average count^d^	3.57 (1.19)	1.93 (1.50)	**< 0.001** ^a^
Unit cost (EUR)	376	376	
Average cost per patient (EUR)^f^	1344	728	
Virtual contact with AirView			
Average count^d^	0.10 (0.34)	0.89 (0.94)	**< 0.001** ^b^
Unit cost (EUR)	162	162	
Average cost per patient (EUR)^f^	16	144	
Virtual contact without AirView			
Average count^d^	0.28 (0.61)	0.58 (1.08)	**< 0.001** ^b^
Unit cost (EUR)	162	162	
Average cost per patient (EUR)^f^	45	94	
Only request for CPAP equipment			
Average count^d^	0.14 (0.43)	1.68 (1.71)	**< 0.001** ^b^
Unit cost (EUR)	162	162	
Average cost per patient (EUR)^f^	23	271	
Total per patient (EUR)^e^	1210 (1129–1614)	1076 (700–1507)	< 0.061^c^

^a^ Tested using a Poisson model adjusted for covariates: age, sex, AHI and ESS.

^b^ Tested using a logistic regression model adjusted for covariates: age, sex, AHI and ESS.

^c^ Tested using linear regression and bootstrap, adjusted for covariates: age, sex, AHI and ESS.

^d^ Mean (SD).

^e^ Median (IQR).

^f^ Per‐patient costs are calculated as (average count × unit cost per contact).

Virtual approaches tend to be more cost‐effective than traditional follow‐up methods (Isetta et al. 2015; Lugo et al. 2019; Turino et al. 2017). In our analysis, we observed no statistically significant difference in overall costs between the two hospitals. The average total cost per patient in Hospital B was 11% lower (134 EUR/patient) compared to Hospital A. This difference may reflect that Hospital A conducted a greater number of in‐person follow‐up contacts, which are more costly, whereas, despite having a greater number of total contacts per patient, Hospital B had less expensive and primarily virtual contacts. These findings are consistent with those of Isetta et al. (2015), who reported similar direct costs between telemedicine‐based and standard face‐to‐face CPAP follow‐up strategies. However, their inclusion of indirect costs, such as savings on transportation, reduced productivity loss, and time saved for patients, led to the conclusion that the telemedicine‐based approach was more cost‐effective overall. Had we included similar indirect costs in our analysis, the virtual follow‐up model would likely have demonstrated even greater cost advantages.

While our study compared virtual and in‐person follow‐up across two hospitals, it does not represent a strict dichotomy between the two approaches. Subsequent follow‐up was predominantly virtual at Hospital B, and primarily in‐person at Hospital A, but some exceptions occurred. The observed variation reflects real‐world clinical practice and supports a representative comparison of these follow‐up models. Importantly, our study included a two‐year follow‐up period, in contrast to the shorter follow‐up durations of 3–6 months reported in studies by Lugo et al. (2019), Turino et al. (2017) and Isetta et al. (2015). This extended follow‐up enhances the reliability of our cost analysis by capturing the long‐term economic implications beyond the initial CPAP initiation phase.

#### 5.3.1 DRG Tariffs

We applied the 2025 DRG tariffs, adjusted to 2022 price levels. A more detailed analysis, based on the time allocation of each healthcare professional group according to type of follow‐up, could potentially have yielded more precise cost estimates. We consider the adjusted DRG tariffs to be a sufficiently robust proxy for resource utilisation in this context. A more time‐specific analysis might have widened the cost differential between in‐person and virtual contacts. Still, it would likely not have altered the overall conclusion: that the scheduled annual in‐person follow‐up visit could be replaced by a more flexible, virtual, and patient‐centred follow‐up model.

Although several studies have demonstrated that telemonitored follow‐up is more cost‐effective than in‐person contacts, this is often based on the assumption that virtual care is less time‐consuming. While this holds true for many virtual follow‐ups, such as those not involving AirView or those requiring equipment delivery, it is important to note that an AirView‐based follow‐up, which includes data review, device programming, and troubleshooting, can be just as time‐intensive as in‐person visits. This nuance should be carefully considered when reallocating resources and redesigning future follow‐up protocols.

### 5.4 Patient Perspective

Recent research has demonstrated that some patients prefer face‐to‐face in‐person contacts, while others favour virtual interactions (Jeppesen, Good, et al. 2025a). Moreover, healthcare professionals need to assess patients' health literacy levels, that is, their ability to understand health‐related information and engage actively with healthcare providers. This is particularly relevant, since health literacy levels have been shown to correlate with patients' capacity for self‐management in chronic diseases (Bo et al. 2014; Mackey et al. 2016). Patients with low health literacy may represent a distinct OSA‐related sociotype that requires a tailored follow‐up approach. Consequently, further research should focus on identifying clinically relevant OSA phenotypes that could benefit from differentiated follow‐up regimens. A more patient‐centred model, incorporating patients' preferences, health literacy, and motivation, may help determine the most appropriate follow‐up modality and identify subgroups who would benefit from more frequent or in‐person contacts.

## 6 Limitations

This study has several limitations that should be acknowledged. First, the smaller‐than‐anticipated number of patients from Hospital A (*n* = 174), as illustrated in Figure 2. This was due to the Hospital's relatively recent adoption of telemedicine‐based CPAP treatment in 2020, which limited the number of patients eligible for inclusion with a complete 2‐year follow‐up. While this reduced the patient size of one hospital, the availability of daily adherence data helps compensate by providing a rich and detailed dataset for analysis, thereby ensuring the reliability of the results.

Second, the non‐randomised design where patients were assigned to follow‐up pathways based on hospital catchment area. This natural allocation introduces the possibility of unmeasured differences between groups, although both hospitals serve broad and comparable populations.

Third, patients at the two hospitals were treated in different calendar periods, with Hospital B starting telemonitoring in 2015 and Hospital A in 2020. This means that patients initiated CPAP in different healthcare contexts and the COVID‐19 pandemic likely influenced access to care, visit modality, and adherence support. This temporal difference introduces potential confounding and should be considered when interpreting the results.

A further limitation is the lack of information on comorbidities, as OSA is frequently associated with a range of comorbid conditions. Uneven distribution of comorbidities between the two hospital populations could potentially bias adherence outcomes or influence follow‐up needs. Access to such data would have enabled a more in‐depth investigation of potential associations with follow‐up phenotypes and should be acknowledged in future studies.

Additionally, nights without CPAP data were coded as zero hours used, and values exceeding 24 h were replaced by the mean of valid observations. While this approach ensures consistency, it may misclassify nights affected by connectivity failures or technical issues. Moreover, CPAP usage data may include periods when the patient is awake, which could affect the interpretation of adherence.

Finally, the study does not include data on patient preferences, motivational factors, or patient‐reported outcomes such as quality of life or satisfaction. Although this is a common limitation in epidemiological research, such information could be valuable in the development of future follow‐up strategies that are more personalised and responsive to individual patient needs.

## 7 Strengths

A strength of the present study is the availability of daily adherence data for each patient over a 2‐year period. When combined with the highly validated data from national Danish registers—covering hospital contacts, procedures, and socioeconomic status—these complementary sources provide a solid basis for gaining broader insights into follow‐up patterns and exploring more detailed analytical perspectives.

The multicentre design, involving sleep clinics from two different regions in Denmark, enhances the generalisability of the findings to the broader Danish OSA population and potentially to other countries with similar healthcare systems. Moreover, the epidemiological design ensures that patients were unaware of their inclusion in the study, thereby reflecting real‐world adherence behaviour. This contrasts with prospective studies, where participants may be more motivated and thus more adherent due to their awareness of the study.

## 8 Perspective and Future Research

Several studies have highlighted that the growing global burden of OSA calls for novel approaches to the follow‐up of patients. Research into OSA phenotypes, including those with comorbidities and other subtypes, is essential to develop tailored follow‐up strategies aimed at achieving and maintaining high adherence (Lyons et al. 2020; Pépin et al. 2024, 2025).

In addition, studies exploring healthcare infrastructure and innovative artificial intelligence (AI)‐based approaches are needed to ensure that the healthcare system is equipped to manage the rising prevalence of OSA. While several studies have already shown promising results using digital interventions to improve CPAP adherence (Labarca et al. 2021; Shaughnessy and Morgenthaler 2019; Aardoom et al. 2020), it is equally important to integrate these solutions effectively into clinical practice and to identify which patients will benefit most from specific technologies.

## 9 Conclusion

This study found no significant difference in CPAP adherence between patients receiving scheduled annual in‐person follow‐up and those offered patient‐initiated virtual contact. Adherence rates were similar in both groups, with 80% of the patients adherent 2 years after CPAP initiation. While Hospital A provided fewer but scheduled in‐person contacts, patients at Hospital B had more frequent, primarily virtual contacts. A cost‐minimisation analysis indicated an 11% reduction (134 EUR/patient) in average overall costs when patient‐initiated virtual follow‐ups were implemented; however, this difference was not statistically significant.

Based on these findings, we suggest that for patients demonstrating high adherence to CPAP therapy within the first 3 months, scheduled in‐person follow‐up visits may be considered for replacement with virtual contacts. More generally, we recommend offering a more individualised and, when relevant, proactive follow‐up strategy, potentially supported by AI‐driven solutions.

## Author Contributions

All authors collaborated on the study’s design. Jeppesen and Kørvel‐Hanquist conducted data collection. Data cleaning was performed by Jeppesen and Wolff. The data analysis was performed by Jeppesen, Wolff, and Petersen. Jeppesen drafted the initial manuscript, and all authors provided critical revisions. All authors reviewed and approved the final version of the manuscript.

## Funding

This work was financially supported by the following grants: Knud and Edith Foundation (62786‐2023), University of Southern Denmark (22/60154), Region of Southern Denmark (22/26264 and 22/33607, ‘Et Sundere Syddanmark’) and University Hospital of Southern Denmark—Hospital Sønderjylland (22/26685). The authors have no financial or technical affiliation with ResMed. CPAP usage data was purchased as raw data from the AirView platform.

## Ethics Statement

The disclosure of patient record information without informed consent was based on a regional council approval according to Section 46, part 2 of the Health Act (22/60970). Approval for collecting data from medical records was obtained from the management of the involved hospitals, and the processing of personal data was notified and approved by the Region of Southern Denmark (23/4511), cf. art 30 of The EU General Data Protection Regulation. The study did not require Ethics Committee approval or informed consent under Danish law for register‐based studies.

## Consent

The authors have nothing to report.

## Conflicts of Interest

The authors declare no conflicts of interest.

## Supporting information


**Data S1:** Sensitivity analysis on the optimal number of weeks post‐intervention for data presentation in the ITSA figure.

**Data S2:** Sensitivity analysis on cost minimisation for total costs.

## Data Availability

The datasets used in this study, which include personal information, are not publicly available, in accordance with Danish law. The data extracted from the registers can be accessed with authorised permission from the national Danish registers. Local, anonymised data is stored at the Sleep Clinic at Zealand University Hospital in Køge and the University Hospital of Southern Denmark in Sønderborg, Denmark, and can be accessed upon a reasonable request. CPAP usage data was purchased from the cloud‐based storage platform ResMed AirView.
