# Supplementary material for: Annual Versus Patient‐Initiated Follow‐Up in Sleep Apnoea Care: A Quasi‐Experimental Study
Source: J Sleep Res. 2026 May 20;35(5):e70350. doi: 10.1111/jsr.70350 (PMC13619034; doi:10.1111/jsr.70350)
Supplement: Supplementary file 1 [file JSR-35-e70350-s001.docx]

**Supporting Information S2: Sensitivity analysis on cost minimisation for total costs**

|  | | Hospital A^a^ | Hospital B^a^ | Test* |
| --- | --- | --- | --- | --- |
| Total per patient – EUR – 20% | 1411 (486) | | 1133 (677) | **<0.000** |
| Total per patient – EUR – 10% | 1419 (493) | | 1184 (700) | **0.009** |
| Total per patient – EUR – 5% | 1424 (496) | | 1209 (712) | **0.016** |
| Total per patient – EUR – ref. | 1428 (500) | | 1235 (724) | 0.061 |
| Total per patient – EUR + 5% | 1432 (503) | | 1260 (737) | 0.158 |
| Total per patient – EUR + 10% | 1436 (507) | | 1285 (750) | 0.280 |
| Total per patient – EUR + 20% | 1445 (514) | | 1336 (777) | 0.757 |
| Total per patient – EUR + 30% | 1453 (522) | | 1387 (805) | 0.658 |
| Total per patient – EUR + 40% | 1462 (530) | | 1438 (834) | 0.258 |
| Total per patient – EUR + 50% | 1470 (538) | | 1489 (864) | 0.066 |
| Total per patient – EUR + 60% | 1478 (546) | | 1539 (894) | **0.013** |

** Tested using linear regression and bootstrap, adjusted for covariates: age, sex, AHI and ESS*

*^a^ Mean (SD)*

Sensitivity analysis on cost minimisation for total costs if virtual costs were different, the in-person costs are kept the same, but the costs of virtual contacts are tested from minus 20% to plus 60%.
