# Supplementary material for: Annual Versus Patient‐Initiated Follow‐Up in Sleep Apnoea Care: A Quasi‐Experimental Study
Source: J Sleep Res. 2026 May 20;35(5):e70350. doi: 10.1111/jsr.70350 (PMC13619034; doi:10.1111/jsr.70350)
Supplement: Supplementary file 2 [file JSR-35-e70350-s002.docx]

**Supporting Information S1: Sensitivity analysis on the optimal number of weeks post-intervention for data presentation in the ITSA figure**

| Weeks after the intervention | Hospital A population with data n (%) | ITSA possible |
| --- | --- | --- |
| W30 | 128 (83%) | Yes |
| W32 | 118 (76%) | Yes |
| W34 | 105 (68%) | Yes |
| W36 | 96 (62%) | Yes |
| W37 | 88 (57%) | Yes |
| W38 | 73 (47%) | Yes |
| W39 | 60 (39%) | No* |
| W40 | 56 (36%) | No* |
| W41 | 40 (26%) | No* |

** The ITSA cannot be reliably estimated due to insufficient patient data at the end of the observation period, making the analysis infeasible in Stata*

To determine the optimal number of weeks post-intervention for data presentation in the ITSA analysis, we conducted a sensitivity analysis using various time cut-offs. The results remained consistent at both 30 and 37 weeks post-intervention, with no statistically significant differences between the hospitals (p-value at 30 weeks = 0.22). We selected 37 weeks for the final analysis, as data were available for more than 50% of the Hospital A population at that time point, making the ITSA feasible. This prevented model instability, as the ITSA could not be conducted at 39 weeks due to insufficient data, since most patients had not reached that length of follow-up.
